# *Taenia* larvae possess distinct acetylcholinesterase profiles with implications for host cholinergic signalling

**DOI:** 10.1371/journal.pntd.0008966

**Published:** 2020-12-21

**Authors:** Anja de Lange, Ulrich Fabien Prodjinotho, Hayley Tomes, Jana Hagen, Brittany-Amber Jacobs, Katherine Smith, William Horsnell, Chummy Sikasunge, Dorit Hockman, Murray E. Selkirk, Clarissa Prazeres da Costa, Joseph Valentino Raimondo

**Affiliations:** 1 Division of Cell Biology, Department of Human Biology and Neuroscience Institute, Faculty of Health Sciences, University of Cape Town, Cape Town, South Africa; 2 Institute for Medical Microbiology, Immunology and Hygiene, Centre for Global Health, Technical University Munich (TUM), Munich, Germany; 3 Department of Life Sciences, Imperial College London, London, United Kingdom; 4 Wellcome Centre for Infectious Diseases Research in Africa, Institute of Infectious Disease and Molecular Medicine and Division of Immunology, Faculty of Health Sciences, University of Cape Town, Cape Town, South Africa; 5 Ludwig Institute for Cancer Research, Nuffield Department of Clinical Medicine, University of Oxford, Oxford, United Kingdom; 6 School of Biosciences, Cardiff University, Cardiff, United Kingdom; 7 Institute of Microbiology and Infection, University of Birmingham, Birmingham, United Kingdom; 8 Laboratory of Experimental and Molecular Immunology and Neurogenetics (INEM), UMR 7355 CNRS-University of Orleans, Orleans, France; 9 School of Veterinary Medicine, Department of Paraclinicals, University of Zambia, Lusaka, Zambia; Uniformed Services University of the Health Sciences, UNITED STATES

## Abstract

Larvae of the cestodes *Taenia solium* and *Taenia crassiceps* infect the central nervous system of humans. *Taenia solium* larvae in the brain cause neurocysticercosis, the leading cause of adult-acquired epilepsy worldwide. Relatively little is understood about how cestode-derived products modulate host neural and immune signalling. Acetylcholinesterases, a class of enzyme that breaks down acetylcholine, are produced by a host of parasitic worms to aid their survival in the host. Acetylcholine is an important signalling molecule in both the human nervous and immune systems, with powerful modulatory effects on the excitability of cortical networks. Therefore, it is important to establish whether cestode derived acetylcholinesterases may alter host neuronal cholinergic signalling. Here we make use of multiple techniques to profile acetylcholinesterase activity in different extracts of both *Taenia crassiceps* and *Taenia solium* larvae. We find that the larvae of both species contain substantial acetylcholinesterase activity. However, acetylcholinesterase activity is lower in *Taenia solium* as compared to *Taenia crassiceps* larvae. Further, whilst we observed acetylcholinesterase activity in all fractions of *Taenia crassiceps* larvae, including on the membrane surface and in the excreted/secreted extracts, we could not identify acetylcholinesterases on the membrane surface or in the excreted/secreted extracts of *Taenia solium* larvae. Bioinformatic analysis revealed conservation of the functional protein domains in the *Taenia solium* acetylcholinesterases, when compared to the homologous human sequence. Finally, using whole-cell patch clamp recordings in rat hippocampal brain slice cultures, we demonstrate that *Taenia* larval derived acetylcholinesterases can break down acetylcholine at a concentration which induces changes in neuronal signalling. Together, these findings highlight the possibility that *Taenia* larval acetylcholinesterases can interfere with cholinergic signalling in the host, potentially contributing to pathogenesis in neurocysticercosis.

## Author summary

Infection of the human nervous system with larvae of the parasite *Taenia solium* is a significant cause of acquired epilepsy worldwide. Despite this, the precise cellular and molecular mechanisms underlying epileptogenesis in neurocysticercosis remain unclear. Acetylcholinesterases are a family of enzymes widely produced by helminthic parasites. These enzymes facilitate the breakdown of acetylcholine, which is also a major neurotransmitter in the human nervous system. If *T*. *solium* larvae produce acetylcholinesterases, this could potentially disrupt host cholinergic signalling, which may in turn contribute to seizures and epilepsy. We therefore set out to investigate the presence and activity of acetylcholinesterases in *T*. *solium* larvae, as well as in *Taenia crassiceps* larvae, a species commonly used as a model parasite in neurocysticercosis research. We found that both *T*. *crassiceps* and *T*. *solium* larvae produce acetylcholinesterases with substantial activity and that the functional protein domains in the *Taenia solium* acetylcholinesterases have great similarity to those of human acetylcholinesterases. We further demonstrate that the acetylcholinesterase activity in the products of these parasites is sufficient to break down acetylcholine at a concentration which induces changes in neuronal signalling in an *ex vivo* brain slice model. This study provides evidence that *Taenia* larvae produce acetylcholinesterases and that these can potentially interfere with cholinergic signalling in the host and contribute to pathogenesis in neurocysticercosis.

## Introduction

Neurocysticercosis is a human disease which arises when larvae of the cestode *Taenia solium* (*T*. *Solium*) infect the central nervous system [1]. The most common symptom of this infection is the development of epileptic seizures, which occur in 70–90% of symptomatic neurocysticercosis cases [2]. As a result, neurocysticercosis is a major cause of adult-acquired epilepsy worldwide. Neurocysticercosis impacts heavily on the quality of life of those infected, and is also a significant drain on the medical and economic resources of endemic countries [3–5]. Despite the global impact of neurocysticercosis, precisely how cerebral infection with *T*. *solium* relates to the development of seizures remains unclear.

It has been well documented that many parasitic worms of the alimentary tract produce substances that aid them in modulating host responses in ways that benefit the parasite [6–8]. Acetylcholinesterases (AChEs), which catalyse the breakdown of acetylcholine, are one family of enzymes that have been implicated in the modulation of host responses. Helminths widely express membrane-bound forms of AChEs, which are classically associated with the facilitation of rapid acetylcholine signalling to parasite muscle, sensory, and neural structures [9,10]. Some also produce surface-presenting membrane-bound AChEs [11–14], or can actively excrete/secrete AChEs, which may modulate acetylcholine dependent components of the host immune response, play a role in detoxification of ingested cholinesterase inhibitors, or inhibit smooth muscle contraction and mucus and fluid secretion associated with clearance of intestinal parasites [10,15–17].

Acetylcholine is also a major neurotransmitter in the human brain, with powerful effects on the excitability of cortical circuits [18,19]. It is a critical component of multiple brain systems that are responsible for functions such as attention, learning, memory, sleep and motor activity [20,21]. Disruption of cholinergic signalling is well known to lead to seizures. For instance, mutations of the nicotinic acetylcholine receptor (in the genes coding for the β4 or α2 subunits) underlie a heritable form of epilepsy called autosomal dominant nocturnal frontal lobe epilepsy [22]. The mutant receptors are more sensitive to acetylcholine than normal receptors, and may generate seizures by promoting and synchronizing spontaneous oscillations in thalamo-cortical circuits [22]. Further, pilocarpine (an acetylcholine muscarinic receptor agonist) is a well described proconvulsant agent [23] and the blockade of endogenous brain AChEs by organophosphate pesticides or poisons can also cause seizures [24,25].

Since *T*. *solium* larvae invade the central nervous system in neurocysticercosis, it is important to determine potential AChE activity expressed by these larvae, as such activity could conceivably interfere with endogenous cholinergic signalling in the brain by breaking acetylcholine down into neurologically inactive products [26]. *Taenia crassiceps* (*T*. *crassiceps*) is a related cestode, which has also been known to invade the human nervous system, and is widely utilised as a model parasite for *T*. *solium* in animal models of cysticercosis and neurocysticercosis [27–29]. It is therefore also important to ascertain how AChE activity might compare between the larvae of these two *Taenia* species.

AChEs have been reported in the adult forms of several members of the broader *Taeniidae* family [30–32] as well as in larval stages [9,11,13,33,34]. The AChEs are often associated with the neural structures and parasite tegument of Taeniids, and there is also some suggestion that some Taeniid larvae may release AChEs into the host environment [33]. Studies describing cholinesterases in *T*. *crassiceps* larvae are scarce, with one report of AChEs in the bladder wall of *T*. *crassiceps* [35], and one other study which refers to the presence of “unidentified esterases” in the cystic fluid of *T*. *crassiceps* [36].

The genome of *T*. *solium* has recently been sequenced [37] and bioinformatics have revealed three *T*. *solium* acetylcholinesterase homologs [16]. Whether the acetylcholinesterases are expressed during the larval stage of *T*. *solium*, and if so, what their activities and locations are, is not yet clear. A histological study by Vasantha *et al*. [38] in *T*. *solium* larvae demonstrated AChE staining in neural structures of the larvae. No obvious staining of AChEs on the surface of the larvae is described, apart from positive staining in a few surface nerve endings. We further found one other report of cholinesterase activity in *T*. *solium* larvae, with activity predominantly present in the isolated cyst bladder ([39] cited in [40]).

Therefore, there is an important need for a detailed characterization of AChE activity in the larvae of different *Taenia* species, as well as an investigation into whether larval derived AChEs could conceivably disrupt host neuronal cholinergic signalling. Here, we used multiple techniques to explore AChEs activity in different extracts of both *T*. *crassiceps* and *T*. *solium* larvae. We find that both the larvae of *T*. *crassiceps* and *T*. *solium* contain significant AChE activity, but it is broadly lower in *T*. *solium* as compared to *T*. *crassiceps* larvae. In addition, whilst AChEs were present in all fractions of *T*. *crassiceps* larvae, including the membrane surface and excreted/secreted extracts, we could not identify AChEs on the membrane surface or within the excreted/secreted extracts of *T*. *solium* larvae. Using bioinformatic approaches we show that functional protein domains in the *Taenia solium* acetylcholinesterases are conserved, when compared to the homologous human sequence. Finally, using whole-cell patch clamp recordings in rodent hippocampal brain slice cultures we demonstrate that *Taenia* larval derived AChEs can break down acetylcholine at a concentration which induces changes in neuronal signalling.

## Materials and methods

### Ethics statement

All animal handling, care and procedures were carried out in accordance with South African national guidelines (South African National Standard: The care and use of animals for scientific purposes, 2008) and with approval from the University of Cape Town Animal Ethics Committee (Protocol No: AEC 019/025, AEC 014/035).

### Bioinformatics

The protein sequences for *T*. *solium* and *T*. *saginata* cholinesterase homologues, identified in Tedla *et al*. [16], were downloaded from https://parasite.wormbase.org/ (Accession numbers: TsM_000001700 [Tso1], TsM_000234300 [Tso2], TsM_001220100 [Tso3], TSAs00071g07627m00001 [Tsa]). The human protein sequence was downloaded from NCBI (Accession number NP000656 [Hs]). Protein sequences were aligned using ClustalW in SnapGene (v5.5.5) and the presented alignment prepared with BOXSHADE (https://embnet.vital-it.ch/software/BOX_form.html). Annotations of protein domains were based on those in Tedla *et al*. [16]. The percent identity matrix for the analysed protein sequences was generated using Clustal Omega (https://www.ebi.ac.uk/Tools/msa/clustalo/).

### *Taenia* acquisition, maintenance, and preparation of cyst extracts

#### Acquisition and maintenance of *T*. *crassiceps* larvae

Larvae (ORF strain) were donated by Dr Siddhartha Mahanty (University of Melbourne, Melbourne, Australia) and propagated *in vivo* by serial intraperitoneal infection of 5-8-week-old female C57BL/6 mice. Every 3 months parasites were harvested by peritoneal lavage and washed 6 times in phosphate buffered saline (PBS, 1X, pH 7.4) before further processing.

#### Preparation of *T*. *crassiceps* whole cyst homogenate

Larvae were frozen at -80°C immediately after harvesting. Upon thawing, larvae were suspended in a volume of PBS threefold that of the larvae. A protease inhibitor cocktail was added to this suspension (1% vol/vol, Sigma-Aldrich). The larvae were then homogenised on ice using a glass tissue grinder. The resulting mixture was centrifuged at 3100 *g* for 20 minutes at 4°C. The liquid supernatant (excluding the low density white floating layer) was collected and sterile filtered through a 0.22 μm size filter (Millex-GV syringe filter, Merck). This supernatant was then aliquoted and stored at -80°C until use. This preparation is referred to as “*T*. *crassiceps* whole cyst homogenate”.

#### Preparation of *T*. *crassiceps* cyst membrane and cyst vesicular fluid extracts

After harvesting, washed larvae (+/- 10ml) were placed onto a piece of filter paper (which had been saturated with 1X PBS) in a metal sieve. Cysts were then ruptured using a weighing spatula. The fluid from the ruptured cysts that passed through the filter paper was collected in a beaker and was centrifuged at 3100 *g* for 20 minutes at 4°C, and the supernatant was collected, aliquoted and stored at -80°C until use. This extract is referred to as “*T*. *crassiceps* cyst vesicular fluid”. The fraction of the cysts that remained on the filter paper were scraped off with the weighing spatula and suspended in an equal volume of PBS containing a protease inhibitor cocktail (1% vol/vol, Sigma-Aldrich). This mixture was freeze-thawed once at -80°C, homogenised on ice using a glass tissue grinder, and centrifuged at 3100 *g* for 20 minutes at 4°C. The liquid supernatant was collected, aliquoted, and stored at -80°C until use. This extract is referred to as “*T*. *crassiceps* cyst membrane”.

#### Preparation of *T*. *crassiceps* larval excretory/secretory extracts

After harvesting, washed larvae (+/- 10 ml) were placed in a 50 ml culture flask with 10 ml culture medium (Earle’s Balanced Salt Solution with 5.3 g/L glucose, 1X Glutamax, 50 U/ml penicillin, 50 μg/ml streptomycin, 100 μg/ml gentamicin sulphate and 11.4 U/ml nystatin). Larvae were maintained at 37°C in 5% CO_2_. After 48 hrs the medium was discarded and replaced with 10 ml fresh media. At 20 days *in vitro* (at which point larvae still displayed motility) the culture media was collected, aliquoted, and stored at -80°C until use. For electrophysiology experiments the excretory/secretory extracts were dialysed/buffer exchanged with PBS using an Amicon stirred cell (Merck) with a 3 kDa molecular weight cut-off membrane, in order to remove small molecules that could potentially induce electrophysiological responses that would interfere with the acetylcholine effect (such as glutamate—see [41].

#### Acquisition of *T*. *solium* larvae

Larvae of *T*. *solium* were harvested from the muscles of a heavily infected, freshly slaughtered pig in Lusaka, Zambia. Larvae were removed from the muscle by vigorous shaking and collected in petri dishes containing sterile PBS (1X, pH 7.4).

#### Preparation of *T*. *solium* whole cyst homogenate

After extensive washing with sterile PBS (1X, pH 7.4), larvae were suspended in a volume of PBS threefold that of the larvae, containing phenylmethyl-sulphonyl fluoride (5 mM) and leupeptin (2.5 μM). Larvae were then homogenised using a sterile handheld homogenizer at 4°C. The resulting homogenate was sonicated (4 x 60 s at 20 kHz, 1 mA, with 30 s intervals), gently stirred with a magnetic stirrer (2 hrs at 4°C), and centrifuged at 15 000 *g* for 60 min at 4°C. The liquid supernatant (excluding the low density white floating layer) was collected and sterile filtered through 0.45 μm size filters (Millex-GV syringe filter, Merck). This supernatant was then collected, aliquoted and stored at -80°C until use. This preparation is referred to as “*T*. *solium* whole cyst homogenate”.

#### Preparation of *T*. *solium* cyst vesicular fluid extracts and cyst membrane and scolex

After extensive washing with sterile PBS, larvae were placed in a petri dish and individually ruptured with a sterile needle. The resulting fluid in the petri dish was collected and centrifuged at 15 000 *g* for 60 min at 4°C. The supernatant was then sonicated (4 x 60 s at 20 kHz, 1 mA, with 30 s intervals), phenylmethyl-sulphonyl fluoride (5 mM) and leupeptin (2.5 μM) were added, and the solution was centrifuged a second time at 15,000 *g* for 60 min at 4°C. The supernatant was collected, aliquoted and stored at -80°C until use. This extract is referred to as “*T*. *solium* cyst vesicular fluid”. The remaining parts of the larvae were again extensively washed with PBS and then suspended in an equal volume of PBS containing phenylmethyl-sulphonyl fluoride (5 mM) and leupeptin (2.5 μM). This suspension was again homogenised using a sterile handheld homogenizer at 4°C. The resulting homogenate was sonicated (4 x 60 s at 20 kHz, 1 mA, with 30 s intervals), gently stirred with a magnetic stirrer (2h at 4°C), and centrifuged at 15,000 *g* for 60 min at 4°C. The liquid supernatant (excluding the low density white floating layer) was collected and sterile filtered through 0.45 μm size filters (Millex-GV syringe filter, Merck). This supernatant was then aliquoted and stored at -80°C until use. This extract is referred to as “*T*. *solium* cyst membrane and scolex”.

#### Preparation of *T*. *solium* excretory/secretory extracts

After harvesting, washed larvae were placed into 6 well plates (+/- 15 per well) with 2 ml culture medium (RPMI 1640 with 10 mM HEPES buffer, 100 U/ml penicillin, 100 μg/ml streptomycin, 0.25 μg/ml amphotericin B and 2 mM L-glutamine). Every 24 h, 1 ml of culture medium was collected from each well and replaced with fresh culture medium. Medium from all wells was pooled each day, aliquoted and stored at -80°C. Media collected on days 1, 2, and 3 *in vitro* were pooled, and are referred to as “*T*. *solium* excretory/secretory extracts”.

All *T*. *crassiceps* and *T*. *solium* larval extracts were assessed for protein concentration using a BCA or Bradford protein assay kit (Sigma-Aldrich), respectively.

### Acetylcholinesterase activity and inhibitor sensitivity

AChE activity was determined by the method of Ellman *et al*. [42] at room temperature with 1 mM acetylthiocholine iodide as substrate in the presence of 1 mM 5,5’-dithiobis(2-nitrobenzoic acid) (DTNB) in 100 mM sodium phosphate (pH 7.0). The reaction was monitored by measuring the absorbance at 412 nm, and hydrolysis of acetylthiocholine iodide calculated from the extinction coefficient of DTNB [42]. Activity was expressed as nanomoles of acetylthiocholine hydrolysed per minute per milligram of total protein in each larval extract (nmol min^-1^ mg^-1^). To test the sensitivity of *Taenia* AChEs to different inhibitors, extracts were preincubated with different concentrations of 1,5-bis(4-allyldimethylammoniumphenyl)pentan-3-one dibromide (BW 284c51), tetraisopropyl pyrophosphoramide (iso-OMPA) or eserine salicylate for 20 min at room temperature in Ellman buffer, prior to the addition of 1 mM acetylthiocholine iodide and enzyme activity determination. Each reaction was assayed a minimum of three times. Where AChE activity was reduced to undetectable levels by inhibitors, a residual activity of 0% was allocated on inhibition curves.

### Non-denaturing polyacrylamide gel electrophoresis (PAGE)

Extracts were electrophoresed in Tris-glycine buffer, pH 8.3, through 7.5% polyacrylamide gels in the absence of denaturing and reducing agents. Electrophoresis was performed at 150 V for 3 hrs on ice. Protein staining (Coomassie) was performed on one set of PAGE gels, and specific staining for AChE activity was performed overnight as described by Selkirk and Hussein [43] adapted from the method of Karnovsky and Roots [44]. The maximum volume of each protein extract was loaded (20 μl), to ensure maximal staining. Protein concentrations of the different extracts varied (*T*. *crassiceps*: whole cyst homogenate = 1.9 mg/ml, cyst membrane = 3.4 mg/ml, cyst vesicular fluid = 3.0 mg/ml and excretory/secretory extracts = 1.32 mg/ml; *T*. *solium*: all extracts = 1.5 mg/ml). AChE stains were performed at least three times to ensure reproducibility.

### *In situ* localisation of acetylcholinesterases

To localise *Taenia* AChEs, *Taenia* larvae were submerged in 10% formalin for 60 min, to fix the tissue. Some of the larvae were then stained overnight for AChE activity as described by Selkirk and Hussein [43], and mounted onto slides as whole mounts. A subset of the fixed larvae was embedded in cryo-embedding medium, frozen overnight at -80°C, and cryo-sectioned the following day at 50 μm. The sections were then similarly stained overnight for AChE activity, placed on positively charged slides and dehydrated in graded alcohols before mounting. To assess non-specific staining, in a subset of the whole mount and cryo-section specimens, acetylthiocholine iodide (the substrate) was omitted during the AChE staining procedure. Specimens were imaged using an upright light microscope.

### An *ex vivo* model to examine the effect of *Taenia* AChEs in the context of neurocysticercosis

#### Hippocampal brain slice preparation

Organotypic brain slices were prepared using 6-8-day-old Wistar rats following the protocol originally described by Stoppini *et al*. [45]. Briefly, brains were extracted and swiftly placed in cold (4°C) dissection media consisting of Earle’s Balanced Salt Solution (Sigma-Aldrich) supplemented with D-glucose (6.1 g/L) and HEPES (6.6 g/L). The hemispheres were separated, and individual hippocampi were removed and immediately cut into 350 μm slices using a Mcllwain tissue chopper (Mickle). Cold dissection media was used to separate and rinse the slices before placing them onto Millicell-CM membranes (Sigma-Aldrich). Slices were maintained in culture medium consisting of 25% (vol/vol) Earle’s balanced salt solution; 49% (vol/vol) minimum essential medium (Sigma-Aldrich); 25% (vol/vol) heat-inactivated horse serum (Sigma-Aldrich); 1% (vol/vol) B27 (Invitrogen, Life Technologies) and 6.2 g/l D-glucose (Sigma-Aldrich). Slices were incubated in a 5% carbon dioxide (CO_2_), humidified incubator at 37°C. Recordings were made after 6–14 days in culture.

#### Electrophysiology

Brain slices were transferred to a submerged recording chamber on a patch clamp rig, which was maintained at a temperature between 28 and 34°C, and were continuously superfused with standard artificial cerebrospinal fluid (120 mM NaCl, 3mM KCl, 2 mM MgCl_2_, 2 mM CaCl_2_, 1.2 mM NaH_2_PO_4,_ 23 mM NaHCO_3_ and 11 mM D-Glucose in deionised water with pH adjusted to between 7.35–7.40 using 0.1 mM NaOH) bubbled with carbogen gas (95% O_2_: 5% CO_2_) using peristaltic pumps (Watson-Marlow). Micropipettes were prepared (tip resistance between 3 and 7 MΩ) from borosilicate glass capillaries (outer diameter 1.2 mm, inner diameter 0.69 mm) (Harvard Apparatus Ltd) using a horizontal puller (Sutter). Micropipettes utilised for whole cell patch clamping were filled with an artificial cell internal solution (126 mM K-gluconate, 4 mM KCl, 10 mM HEPES, 4 mM Na_2_ATP, 0.3 mM NaGTP and 10 mM Na_2_-phosphocreatine) before being placed over the recording electrode.

Neurons in the CA3 region of the hippocampus were visualized using an upright microscope with a 20X water immersion objective. Surface cells with a typical pyramidal cell body morphology were selected for whole cell patching. To explore the ability of *Taenia* acetylcholinesterase activity to alter neuronal acetylcholine signalling, two additional “puffer” micropipettes were lowered to the cell surface once a neuron had been patched. One of these micropipettes contained a solution of 200 μm acetylcholine with 1.3 mg/ml *T*. *crassiceps* excretory/secretory extracts, while the second contained a solution of 200 μm acetylcholine with 1.3 mg/ml *T*. *crassiceps* excretory/secretory extracts that had been heated to 56°C for 30 min to inactivate enzymes. Current was injected to hold the membrane potential of cells close to their action potential firing threshold. Five 30 ms puffs (~20 psi) of one of the solutions was then applied to the cell’s surface using an OpenSpritzer [46] and the neuron’s response recorded for 26 s before a 94 s “recovery” period was allowed. Thereafter an identical puff train of the other solution was applied, and the neuron’s response again recorded for 26 s. After another 94 s recovery period, the cycle was repeated.

Post-recording analysis consisted of counting the number of action potentials induced by the application of each solution within a 5 s period of the onset of the puff train. Only traces with a baseline membrane potential prior to the puff application of between -60 mV and -45 mV were included. Each data point in the puffing experiments represents the average of between 2 and 5 repeats of the puff cycle. Matlab (MathWorks) was utilised for trace analysis.

### Data analysis and statistics

Data was visualised and analysed using Matlab, Microsoft Excel and GraphPad Prism. Each dataset was subjected to a Shapiro-Wilk test to determine whether it was normally distributed. Most datasets proved to be non-normal and as such non-parametric analyses were utilised throughout. These included: Kruskal-Wallis analyses with Dunn’s multiple comparison post-hoc tests and Mann-Whitney tests. The confidence interval for all tests was set at 95%.

## Results

### All *T*. *crassiceps* larval extracts display acetylcholinesterase activity

**Fig 1:**
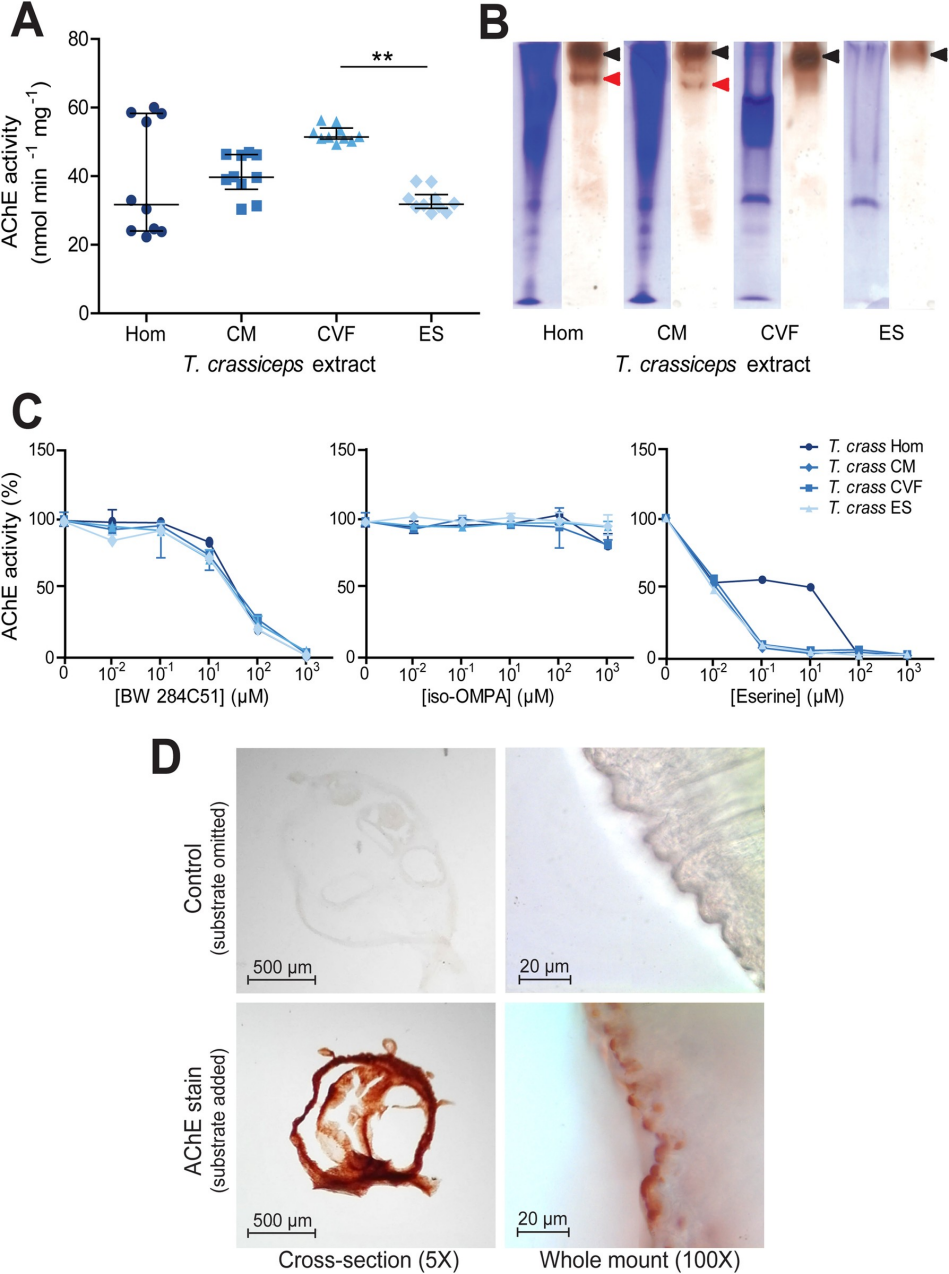
Identification and characterisation of acetylcholinesterases in *Taenia crassiceps* larval extracts. A) Quantification of acetylcholinesterase (AChE) activity in different *Taenia crassiceps* larval extracts. AChE activity was quantified using the method of Ellman *et al*. [42] with 1 mM acetylthiocholine iodide as substrate in the presence of 1 mM 5,5’-dithiobis(2-nitrobenzoic acid) in 100 mM sodium phosphate, pH 7.0, at room temperature. The extracts assessed were: whole cyst homogenate (Hom); cyst membrane (CM), cyst vesicular fluid (CVF) and larval excretory/secretory extracts (ES). Values with median ± IQR, N = 10 for all extracts assayed, **p ≤ 0.01, Kruskal-Wallis test with Dunn’s multiple comparison post-hoc tests B) Non-denaturing polyacrylamide gel electrophoresis of *Taenia crassiceps* extracts. Extracts were electrophoresed in Tris-glycine buffer, pH 8.3, through 7.5% polyacrylamide gels in the absence of denaturing and reducing agents. Coomassie staining was performed on one set of gels (left tracks), and staining for AChE activity [44] was performed on another set of gels for 16 hrs after incubation with the substrate (right tracks). The maximum volume of each extract was loaded (20 μl), to ensure maximal staining. Protein concentrations of the different extracts varied: Hom = 1.9 mg ml^-1^, CM = 3.4 mg ml^-1^, CVF = 3.0 mg ml^-1^ and ES = 1.32 mg ml^-1^. C) Inhibitor sensitivity of *Taenia crassiceps* AChEs. *Taenia crassiceps* extracts were preincubated with BW 284C51, iso-OMPA or eserine salicylate (Eserine) for 20 min at room temperature in Ellman buffer, prior to the addition of 1 mM acetylthiocholine iodide and enzyme activity determination. Median ± Range, N = 10 for all extracts in absence of inhibitors, N = 3 for all extracts at all inhibitor concentrations. D) Localisation of larval AChEs. Cryo-sections (N = 19 for control and N = 19 for AChE stain) and whole mounts (N = 15 for control and N = 15 for AChE stain) of *Taenia crassiceps* larvae were subjected to AChE staining [44] for 16 hrs prior to dehydration and mounting. Images in top panels show time-matched controls where acetylthiocholine iodide was omitted from the staining solution. Cross sections were imaged at 5X magnification and whole mounts were imaged at 100X magnification.

In order to quantify AChE activity in *T*. *crassiceps* larvae, Ellman’s assays were employed, using acetylthiocholine as a substrate [42]. These assays revealed that all *T*. *crassiceps* larval extracts had significant AChE activity (**Table 1**and **Fig 1A**). A Kruskal Wallis one-way ANOVA with post hoc Dunn’s Multiple Comparison tests revealed that the only statistically significant difference between the median activities of the different *T*. *crassiceps* larval extracts was between that of the cyst vesicular fluid and that of the excretory/secretory extracts (P ≤ 0.01, **Fig 1A**).

**Table 1:** Acetylcholinesterase (AChE) activity of different larval extracts of T. crassiceps and T. solium.

Larval species	Larval extract	# of assays	Median AChE activity (nmol min^-1^ mg^-1^)
	Whole cyst homogenate	10	31.7 (IQR 24.0–58.3)
*Taenia crassiceps*	Cyst membrane	10	39.7 (IQR 36.1–46.3)
	Cyst vesicular fluid	10	51.5 (IQR 50.9–54.1)
	Excretory/secretory extracts	10	31.8 (IQR 50.9–54.1)
	Whole cyst homogenate	5	4.1 (IQR 4.1–5.1)
*Taenia solium*	Cyst membrane & scolex	5	14.0 (IQR 13.8–15.3)
	Cyst vesicular fluid	5	4.7 (IQR 3.37–5.03)
	Excretory/secretory extracts	4	Undetectable

To visually confirm *T*. *crassiceps* AChE activity, and to assess whether there may be more than one AChE isoform in *T*. *crassiceps* larval extracts, non-denaturing PAGE gels were run, and stained for AChE. A second set of non-denaturing PAGE gels were run simultaneously and Coomassie stained. These demonstrated that the different *T*. *crassiceps* larval extracts contained different protein compositions (left tracks in **Fig 1B**). The AChE stained gels (right track for each larval extract in **Fig 1B**) showed distinct dark bands (indicated by black arrowheads) in the tracks of all the *T*. *crassiceps* larval extracts, thereby confirming that all the *T*. *crassiceps* larval extracts show AChE activity. In the whole cyst homogenate and the cyst membrane tracks of the AChE stained gels, there was an additional smaller band (indicated by the red arrowheads in **Fig 1B**). These results suggest that *T*. *crassiceps* larvae express more than one isoform of AChE.

### Inhibitor sensitivity of *T*. *crassiceps* larval acetylcholinesterases

The sensitivity of AChE activity in *T*. *crassiceps* larval extracts to different inhibitors was tested by preincubating them for 20 min with different concentrations of BW 284c51 (a selective AChE inhibitor), iso-OMPA (a selective butyryl cholinesterase inhibitor) or eserine salicylate (a nonselective cholinesterase inhibitor) before assaying AChE activity [42]. All *T*. *crassiceps* extracts showed a similar dose-dependent inhibitory response to BW 284c51, with AChE activity in all extracts being almost completely inhibited by the presence of 1000 μM BW 284c51 (**Fig 1C and S1 Table**). Conversely, the AChE activity of *T*. *crassiceps* extracts was not greatly inhibited by iso-OMPA, with only very small reductions in activity being observed even at high (1 mM) inhibitor concentration (**Fig 1C and S1 Table**). AChE activity in *T*. *crassiceps* cyst membrane, cyst vesicular fluid and excretory/secretory extracts was highly sensitive to eserine inhibition, with strong inhibition apparent at low (1 μM) eserine concentration (**Fig 1C and S1 Table**). The AChE activity of *T*. *crassiceps* whole cyst homogenate was less sensitive to eserine inhibition, only displaying strong inhibition at a much higher eserine concentration (100 μM) (**Fig 1C and S1 Table)**. These inhibition patterns suggest that the cholinesterase produced by *T*. *crassiceps* larvae can be classified as true AChEs, as opposed to pseudocholinesterases [47].

### *T*. *crassiceps* acetylcholinesterases are ubiquitous within the tegument membrane and are present on the larval surface

To spatially localise AChEs within the larvae, both cross-sections of larvae and whole larvae were subjected to AChE staining [44], (**Fig 1D**). To evaluate non-specific staining, a second set of larval cross-sections and whole larvae were subjected to the same staining procedure, with the exception that the substrate (acetylthiocholine iodide) was omitted. Samples where the substrate was omitted (**top panels, Fig 1D**) showed minimal staining. In contrast, cross sections stained for AChE activity displayed dense, uniform staining, indicating that AChEs are localised ubiquitously throughout the tegument membrane (**bottom-left panel, Fig 1D**). Whole larvae stained for AChE revealed that staining localised to numerous small protrusions on the surface of the cyst tegument membrane (**bottom-right panel, Fig 1D**).

### *T*. *solium* larvae produce acetylcholinesterases but do not actively excrete/secrete them

**Fig 2:**
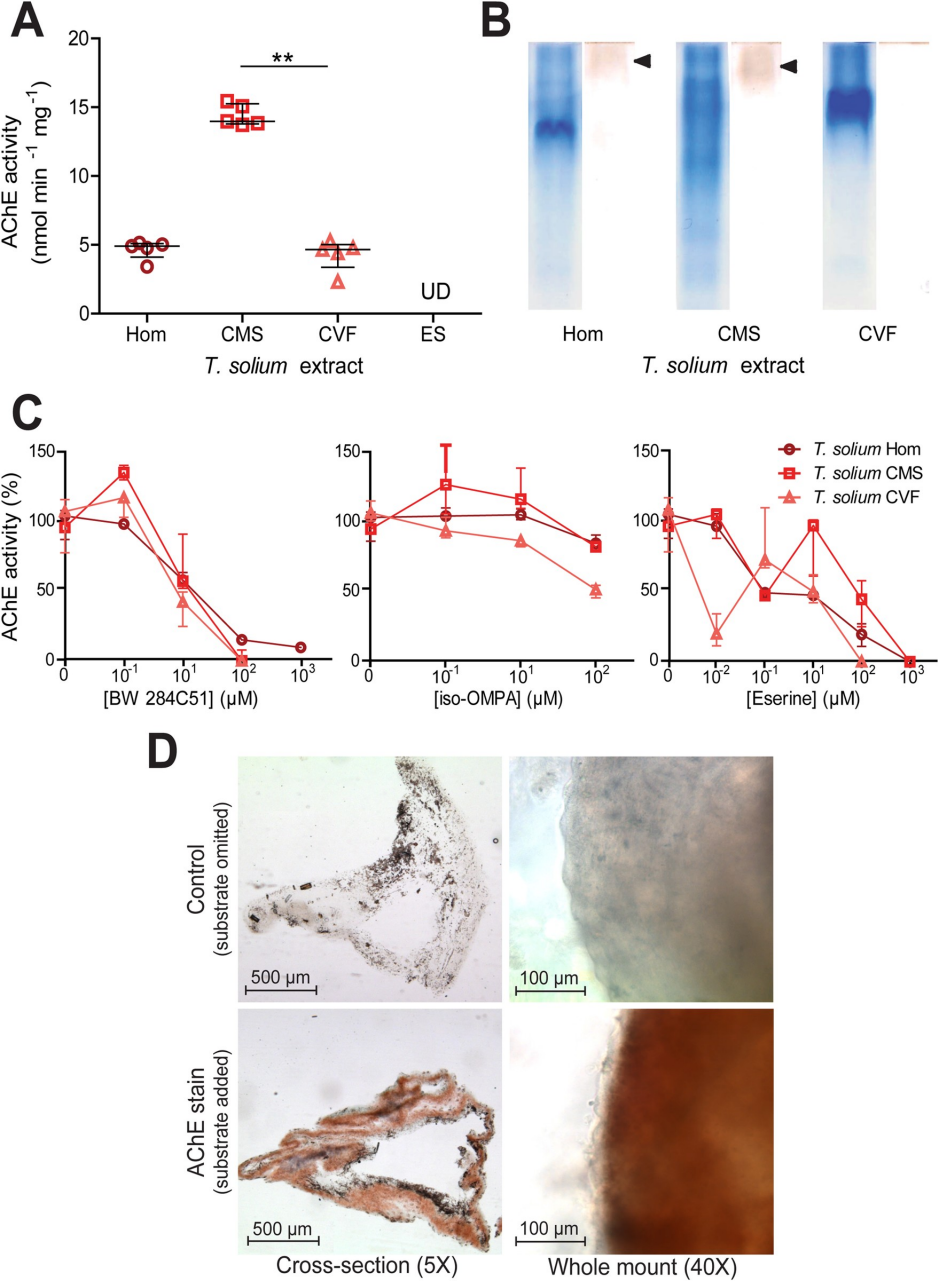
Identification and characterisation of acetylcholinesterases in *Taenia solium* extracts. A) Quantification of acetylcholinesterase (AChE) activity in different *Taenia solium* extracts as in **Fig 1**. The extracts assessed were: whole cyst homogenate (Hom); cyst membrane and scolex (CMS), cyst vesicular fluid (CVF) and larval excretory/secretory extracts (ES). Values with median ± IQR, N = 5 for all extracts assayed, **p ≤ 0.01, Kruskal-Wallis test with Dunn’s multiple comparison post-hoc tests. UD = undetectable. B) Non-denaturing polyacrylamide gel electrophoresis of different *Taenia solium* extracts. Coomassie staining was performed on one set of PAGE gels (left tracks), and staining for AChE activity [44] was performed on another set of gels (right tracks). For each extract, 30 μg of total protein was loaded. C) Inhibitor sensitivity of *Taenia solium* AChEs. *Taenia solium* extracts were preincubated with BW 284C51, iso-OMPA or eserine salicylate (Eserine) for 20 min at room temperature in Ellman buffer, prior to the addition of 1 mM acetylthiocholine iodide and enzyme activity determination. Median ± Range, N = 5 for all extracts in absence of inhibitors and at 10 μM inhibitor concentration, N = 3 for all extracts at all other inhibitor concentrations. D) Localisation of larval AChEs. Cryo-sections (N = 20 for control and N = 20 for AChE stain) and whole mounts (N = 4 for control and N = 4 for AChE stain) of *Taenia solium* larvae were subjected to AChE staining [44]. Images in top row show time-matched controls where acetylthiocholine iodide was omitted from the staining solution. Cross sections were imaged at 5X magnification and whole mounts were imaged at 40X magnification.

Next, we focussed on the major pathogenic cestode of humans; *T*. *solium*. Ellman’s assays revealed that *T*. *solium* whole cyst homogenate, cyst membrane and scolex, and cyst vesicular fluid had detectable AChE activity, whilst the excretory/secretory extracts consistently displayed no detectable AChE activity (**Table 1 and Fig 2A**). A Kruskal Wallis one-way ANOVA with post hoc Dunn’s Multiple Comparison tests revealed that the cyst membrane and scolex had a statistically significantly higher activity than that of the whole cyst homogenate and the cyst vesicular fluid, whilst the median activity of two latter extracts did not differ significantly from one another (P ≤ 0.01, **Fig 2A**).

To visually confirm AChE activity in *T*. *solium*, and to assess whether there may be more than one AChE isoform in larval extracts, non-denaturing PAGE gels were resolved and stained for AChE (**Fig 2B**). Again, a second set of non-denaturing PAGE gels were run simultaneously and Coomassie stained. The Coomassie stain showed that each extract has a distinct protein profile composition (left tracks in **Fig 2B**). The AChE stained gels (right track for each larval extract in **Fig 1B**) revealed bands in the whole cyst homogenate and in the cyst membrane and scolex preparations (indicated by black arrowheads), but no apparent band in the cyst vesicular fluid track. The absence of bands in the cyst vesicular fluid tract, and the relatively faint bands in tracks of the other extracts can be attributed to the fact that the amount and concentrations of the extracts loaded contain a drastically lower total AChE activity than is usually recommended for this technique [43]. In future investigations this could be solved by concentration or purification of the AChEs in *T*. *solium* extracts.

### Inhibitor sensitivity of *T*. *solium* larval acetylcholinesterases

The sensitivity of AChE activity in *T*. *solium* larval extracts to different inhibitors was tested by preincubating them for 20 min with different concentrations of BW 284c51, iso-OMPA or eserine salicylate, before assaying AChE activity [42]. All *T*. *solium* extracts showed a similar dose-dependent inhibitory response to BW 284c51, although the *T*. *solium* whole cyst homogenate appears somewhat less sensitive to inhibition than the cyst membrane and scolex and cyst vesicular fluid (**Fig 2C and S2 Table**). *T*. *solium* extracts showed low sensitivity to inhibition by iso-OMPA (**Fig 2C and S2 Table**). *T*. *solium* whole cyst homogenate, cyst membrane and scolex, and cyst vesicular fluid showed very variable sensitivities to inhibition by increasing concentrations of eserine, but were ultimately all strongly inhibited at an eserine concentration of 1 mM or less (**Fig 2C and S2 Table**). These inhibition patterns suggest that the cholinesterases produced by *T*. *solium* larvae can be classified as true AChEs, as opposed to pseudocholinesterases [47].

### *T*. *solium* larval acetylcholinesterases are localised within the cyst tegument membrane, but do not appear to present on the surface of the parasite

Next, we set out to spatially localise AChEs within the larvae. To do so both cross-sections of larvae and whole larvae were subjected to the same AChE staining as was applied to *T*. *crassiceps* larvae. Control samples where the substrate was omitted to evaluate non-specific staining (top panels, **Fig 2D**), showed some patchy black background staining. AChE stained cross-sections showed uniform, although not very dense, AChE staining throughout the tegument membrane (in addition to the black background staining) (bottom-left panel, **Fig 2D**). High magnification images of the surface of the tegument membrane in whole-mounted AChE-stained larvae revealed that, unlike in *T*. *crassiceps*, AChEs in the tegument membrane of *T*. *solium* are not surface-presenting (bottom-right panel, **Fig 2D**).

### Homology of *Taenia solium*, *Taenia saginata* and *Homo sapiens* acetylcholinesterases

**Fig 3:**
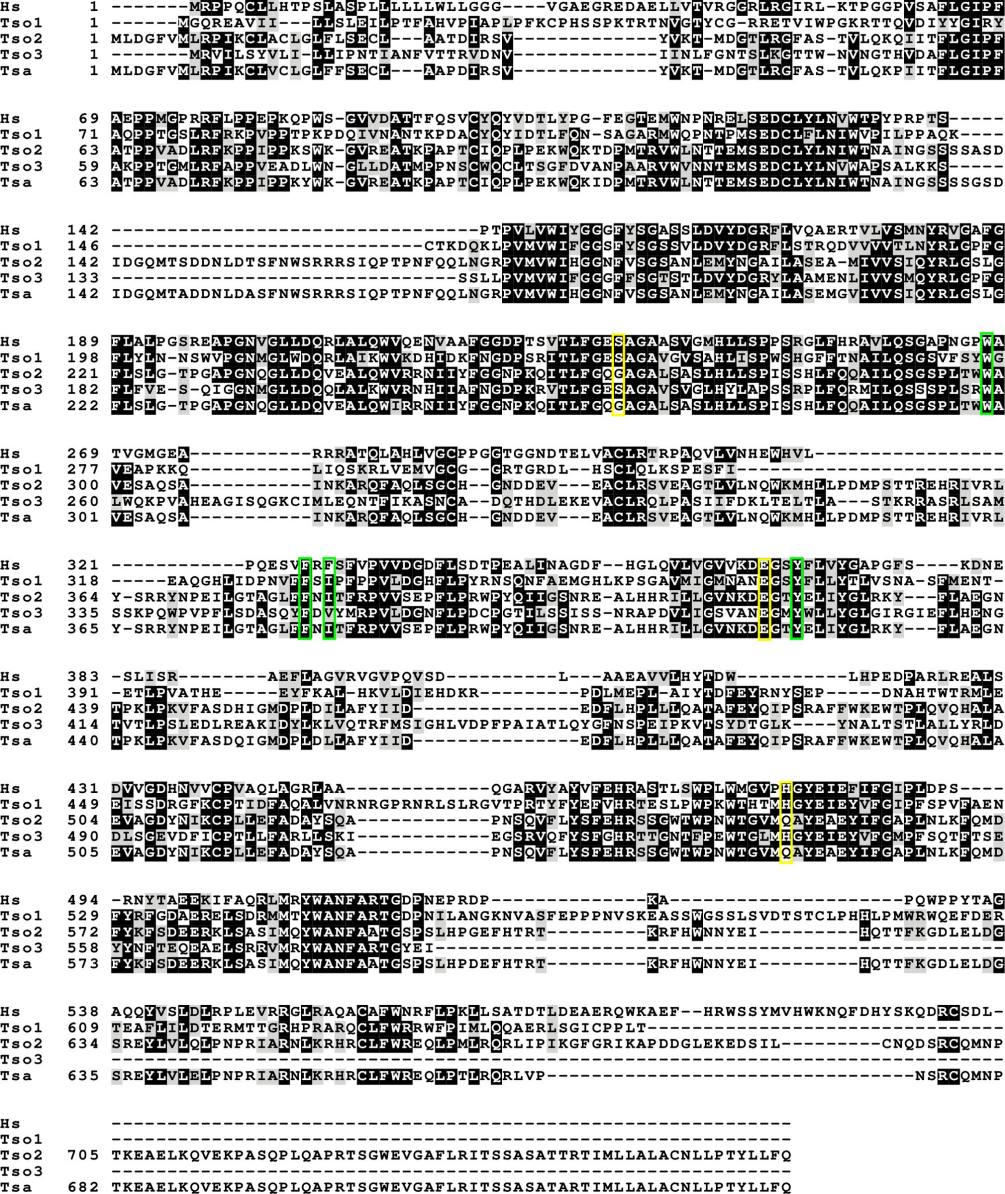
Full amino acid sequence alignment for *Taenia solium*, *Taenia saginata* and *Homo sapiens* acetylcholinesterase proteins. (Accession numbers: NP000656 [Hs], TsM_000001700 [Tso1], TsM_000234300 [Tso2], TsM_001220100 [Tso3], TSAs00071g07627m00001 [Tsa]). The catalytic triad is indicated in yellow and the acyl binding pocket is indicated in green.

Given that the genome for *T*. *solium* and the related species *Taenia saginata* (*T*. *saginata*) are available (the genome for *T*. *crassiceps* has not yet been sequenced), we performed amino acid sequence alignments to determine the putative structural resemblance between host (*Homo sapiens*) and parasite acetylcholinesterase enzymes. The analysis of full amino acid sequence alignments for *T*. *solium*, *T*. *saginata* and *Homo sapiens (H*. *sapiens*) acetylcholinesterases (**Fig 3**) revealed the percent identity between the acetylcholinesterase of *T*. *saginata* (TSa_s00071g07627m00001) and one of the *T*. *solium* acetylcholinesterase sequences (Ts_000234300) to be 97%, whilst there was only a 33% and 38% identity between the acetylcholinesterase of *T*. *saginata* and the other two *T*. *solium* acetylcholinesterase sequences (Ts_000001700 and Ts_001220100, respectively). The percent identity between the three different *T*. *solium* acetylcholinesterase sequences ranged from 33% to 38%, and the percent identity of these to the of *H*. *sapiens* acetylcholinesterase (Hs_NP_000656) similarly ranged from 36% to 39%. Importantly, the “catalytic triad”, which has been described to form the active site for ester hydrolysis within the enzyme, is fully conserved between *H*. *sapiens* and two of the three *T*. *solium* acetylcholinesterase amino acid sequences (TsM 000001700 [Tso1], and TsM 001220100 [Tso3], yellow boxes in **Fig 3**), while three of the four sites that form the “acyl binding pocket”, a region of the enzyme that confers substrate specificity, are fully conserved across all the analysed sequences (green boxes in **Fig 3**) [16,48]. These findings support our earlier findings that *T*. *solium* contains cholinesterases and that these are true acetylcholinesterases, as opposed to pseudocholinesterases.

### Larvae of *T*. *solium* have less acetylcholinesterase activity as compared to *T*. *crassiceps*, and *T*. *crassiceps* larvae excrete/secrete acetylcholinesterases, whilst *T*. *solium* larvae do not

Comparison of AChE activity in the extracts of *T*. *crassiceps* versus the comparable *T*. *solium* extracts show that *T*. *solium* extracts consistently have significantly lower AChE activities than those of *T*. *crassiceps* (P ≤ 0.001, Mann Whitney tests, **Table 1**). Further, a different pattern of AChE distribution within the cyst is observed between the two species–*T*. *solium* AChEs appear to be predominantly located in the cyst membrane and scolex, whilst *T*. *crassiceps* AChEs appear abundant in both the cyst membrane and in the cyst vesicular fluid and are additionally excreted/secreted (**Table 1**). *T*. *crassiceps* also displays surface presenting AChEs, whilst *T*. *solium* do not, as revealed by AChE staining of whole larvae (**Figs 1D and 2D**). It is also noteworthy that *T*. *crassiceps* and *T*. *solium* larval extracts display different inhibitor sensitivities to BW 284c51, iso-OMPA and particularly to eserine (**Fig 1C** versus **Fig 2C**). This suggests that the two parasites produce different forms of AChE, although further investigation would be required to confirm this.

### *Taenia* larval acetylcholinesterases have sufficient activity to break down acetylcholine at a concentration which induces changes in neuronal signalling in an *ex vivo* brain slice model

**Fig 4:**
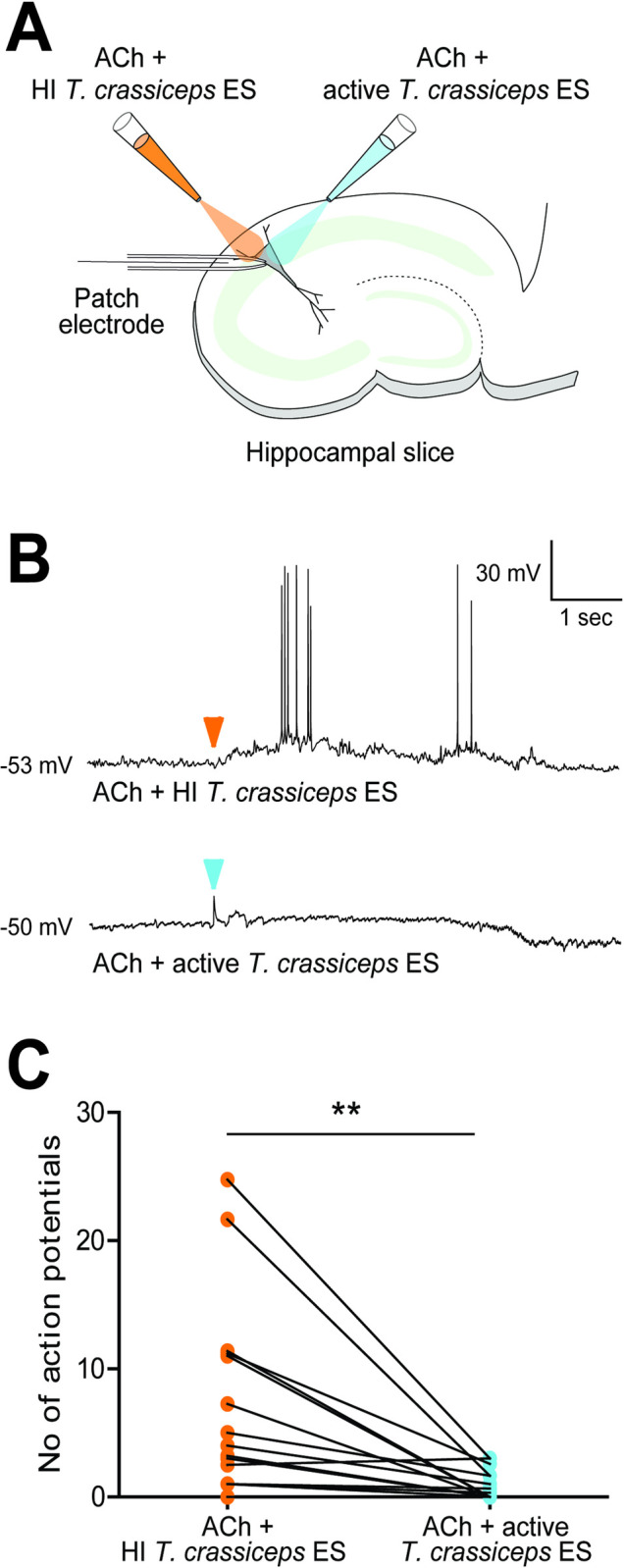
*Taenia* acetylcholinesterases can break down acetylcholine at a concentration which induces changes in neuronal signalling. A) Schematic depicting the experimental setup where whole-cell patch-clamp recordings were made from rat CA3 pyramidal neurons in organotypic hippocampal brain slice cultures. Whilst recording the electrical activity from the neurons, two glass pipettes delivered picolitre volumes of acetylcholine (ACh) (200 μM) with heat-inactivated (at 56°C for 30 min) *Taenia crassiceps* excretory/secretory extracts (1.3 mg ml^-1^), or of ACh (200 μM) with unheated/active *Taenia crassiceps* excretory/secretory extracts (1.3 mg ml^-1^). B) The membrane potential responses of a pyramidal neuron when a solution of ACh with heat-inactivated (HI) *Taenia crassiceps* excretory/secretory extracts (ES) (top trace) or of ACh with active *Taenia crassiceps* ES (bottom trace) was puffed onto the cell body (arrowheads indicate moment of application). C) Population data (median ± IQR) where each point represents the mean number of action potentials evoked in 5 s after neurons were exposed to 5 x 30 ms puffs (2–5 repeat cycles) of either a solution of ACh with HI *Taenia crassiceps* ES (N = 13) or of ACh with active *Taenia crassiceps* ES (N = 13). **p ≤ 0.01, Wilcoxon signed-rank test.

In order to investigate what implications the presence of *Taenia* larval AChEs may have in the context of neurocysticercosis, 200 μM acetylcholine (known to induce depolarisation in hippocampal pyramidal neurons [49]) was applied to neurons in hippocampal organotypic cultures, together with either heat-inactivated *T*. *crassiceps* excretory/secretory products, or active *T*. *crassiceps* excretory/secretory products. The response of the membrane potential of the neurons was measured using whole-cell patch-clamp recordings (see Materials and Methods, and **Fig 4A and 4B**). When neurons were held at a voltage close to their action potential threshold and picolitre volumes of 200 μM acetylcholine with heat-inactivated excretory/secretory products were puffed toward the soma of the neurons, they depolarised and fired action potentials (APs) (median = 3.6 APs, IQR = 1.0–11.2 APs, N = 16, **Fig 4B and 4C**). However, when 200 μM acetylcholine with active excretory/secretory products were applied to the same neurons just 2 min prior to/after this, the neurons did not show the same response, often firing no action potentials despite still being held at a voltage close to their action potential threshold (median = 0.2 APs, IQR = 0.0–1.7 APs, N = 16, P = 0.0017, Wilcoxon signed-rank test, **Fig 4C**). Whilst this formally only demonstrates that a heat-labile component of the extract has an inhibitory effect, this is highly likely due to AChEs in the *T*. *crassiceps* larval excretory/secretory products. These experiments therefore indicate that *T*. *crassiceps* larval excretory/secretory products have sufficient AChE activity to break down acetylcholine at a concentration which induces changes in neuronal signalling in this *ex vivo* brain slice model. This would hold true for *T*. *solium* cyst membrane and scolex AChEs, given twice the amount of time, or the use of an extract of double the concentration, as these break down acetylcholine at roughly half the rate of *T*. *crassiceps* larval excretory/secretory extracts (**Table 1**).

## Discussion

Here we have used multiple methods to characterise the amount and spatial localization of AChE activity in larvae of the cestodes *T*. *crassiceps* and *T*. *solium*. Previous studies have identified AChE activities in the larvae of multiple species of the broader Taeniidae family including *Echinococcus granulosus* (dog tapeworm) [30,33,34] and *Taenia pisiformis* (rabbit tapeworm) [13]. To our knowledge our data represent the first definitive measurements of AChE activity from larvae of *T*. *crassiceps*. The amount of AChE activity we report in *T*. *crassiceps* whole cyst homogenate is similar to that previously reported for the larval homogenate of *T*. *pisiformis* (*T*. *crassiceps* whole cyst homogenate median activity = 31.69 nmol min^-1^ mg^-1^, *T*. *pisiformis* homogenate mean activity = 24.8 nmol min^-1^ mg^-1^) [13]. Interestingly we found that whilst *T*. *solium* larvae also exhibit substantial AChE activity, this activity is broadly less than in extracts from *T*. *crassiceps*. Furthermore, the spatial profile of larval AChE activity is different between these two species. Whilst AChEs were present in all fractions of *T*. *crassiceps* larvae, and presented on the tegument membrane surface, we could not identify AChEs on the tegument membrane surface or within the excreted/secreted extracts of *T*. *solium* larvae. The lack of surface staining in *T*. *solium* larvae is in accordance with a previous study by Vasantha *et al*. (1992), which reports AChEs in *T*. *solium* larvae to be associated with a sub-tegumental network of nerves in the strobila and bladder wall.

Our findings have important implications in the context of *T*. *crassiceps* being utilised as a model organism for neurocysticercosis research, where both larval extracts and whole early-stage cysts of *T*. *crassiceps* have been employed [50–53]. Our data illustrate that there exist significant differences in the cholinergic biology of *T*. *solium* and species that have been utilised as model parasite for *T*. *solium*, such as *T*. *crassiceps*. Once the genome for *T*. *crassiceps* has been sequenced, future work will also be able to determine potential differences in AChE amino-acid sequences between cestode species. Notably, the reported AChE activity of tetrathyridia of *Mesocestoides corti*, another popular model parasite for neurocysticercosis research, is far greater than that which we report for both *T*. *crassiceps* and *T*. *solium* [54]. These differences should be considered when selecting an appropriate model parasite for NCC research.

Our observation that *T*. *solium* larvae do not excrete/secrete AChEs is interesting, as many helminth species have been observed to secrete these enzymes in substantial amounts, with proposed benefits to parasite survival, such as protection against ingested AChE inhibitors and modulation of the host immune response [7,10,16]. Recently, a study by Vaux *et al*. (2016) demonstrated that *in vivo* exposure to secreted AChE from *Nippostrongylus brasiliensis* promoted classical activation of macrophages (as opposed to alternative activation), a state which is permissive to the survival of parasitic nematodes. In contrast, classically activated macrophages appear to be deleterious to the survival of Taeniid larvae, occurring in the resistant Th1 acute phase of infection, whilst their phenotype is shifted to an alternatively activated state during chronic Taeniid infection [8,55]. This could potentially explain why it may not be beneficial for Taeniids to secrete large amounts of AChE.

Our bioinformatics analysis demonstrated that there was modest overall sequence identity between *T*. *solium* and *H*. *sapiens* acetylcholinesterase sequences, as has been previously reported for *Schistosoma mansoni* [16]. However, the fact that protein functional domains appear to be conserved between *T*. *Solium* and *H*. *sapiens* lends support to our experimental findings that *Taenia* larvae contain active AChEs. In addition, using whole-cell patch clamp recordings in rodent hippocampal brain slice cultures, we show that *Taenia* larval-derived AChEs have sufficient activity to break down acetylcholine at a concentration which induces changes in neuronal signalling. These experiments demonstrate that although the activities of *Taenia* larval-derived AChEs may not seem substantial when compared to that of other parasitic worms, such as *Nippostrongylus brasiliensis* (which has a reported excretory/secretory extract AChE activity of 5.3 μmol min^-1^ mg^-1^ [56]), they may well be substantial enough to exert an effect in the sensitive brain environment. It should also be taken into consideration that in clinical presentations of NCC, *T*. *solium* larvae interface directly with brain tissue, which means that the membrane-bound AChEs in *T*. *solium* larvae identified in this study could potentially have a direct and spatially concentrated impact on brain tissue when the cyst starts to degrade and lose membrane integrity. Although we have demonstrated in this study that larval-derived AChEs have sufficient activity to break down acetylcholine at a concentration which induces changes in neuronal signalling in an *ex vivo* brain slice model, further electrophysiological investigations, preferably using purified *T*. *solium* AChE applied in an *in vivo* model, are required to determine whether the activity of larval-derived AChEs can, in fact, functionally alter neuronal signalling.

The cause of seizures and epilepsy secondary to neurocysticercosis has been a matter of great controversy, with some researchers questioning whether neurocysticercosis is truly causative of epilepsy, and with different studies sometimes reporting contrasting results [57–59]. One thing that has become increasingly clear in recent years, however, is that inflammation is closely linked to the generation of seizures and epilepsy, both generally, and within the context of neurocysticercosis [60,61]. In the healthy brain, microglia and astrocytes regulate inflammatory signalling in the brain via, amongst others, acetylcholine receptor dependent signalling [62]. Activation of acetylcholine receptors has been shown to strikingly impair acute phase inflammation [63,64]. One might hypothesise, then, that exposure of the brain to *T*. *solium* membrane AChEs during cyst degeneration could exacerbate perilesional inflammation if the larval AChEs break down acetylcholine molecules involved in anti-inflammatory signalling. Based on this supposition, future investigations involving the addition of AChE inhibitors to NCC model systems that present with severe perilesional inflammation, could be of potential value.

In summary, our findings describe distinct profiles of acetylcholinesterase activity in *T*. *crassiceps* and *T*. *solium* larvae. In light of this, we encourage neurocysticercosis researchers to take into consideration that differences do exist between *T*. *solium* and related cestodes such as *T*. *crassiceps*. We also highlight the possibility of larval-derived acetylcholinesterases interfering with host neural and immune signalling in the brain.

## Supporting information

S1 Table Sensitivity of cholinesterase activity of T. crassiceps extracts to different inhibitors. Legend: UD = undetectable, where activity was so low as to be undetectable.
(TIF)

S2 Table Sensitivity of cholinesterase activity of T. solium extracts to different inhibitors. Legend: UD = undetectable, where activity was so low as to be undetectable.
(TIF)
