# Supplementary figures and images for: Taenia larvae possess distinct acetylcholinesterase profiles with implications for host cholinergic signalling
Source: PLoS Negl Trop Dis. 2020 Dec 21;14(12):e0008966. doi: 10.1371/journal.pntd.0008966 (PMC7785214; doi:10.1371/journal.pntd.0008966)

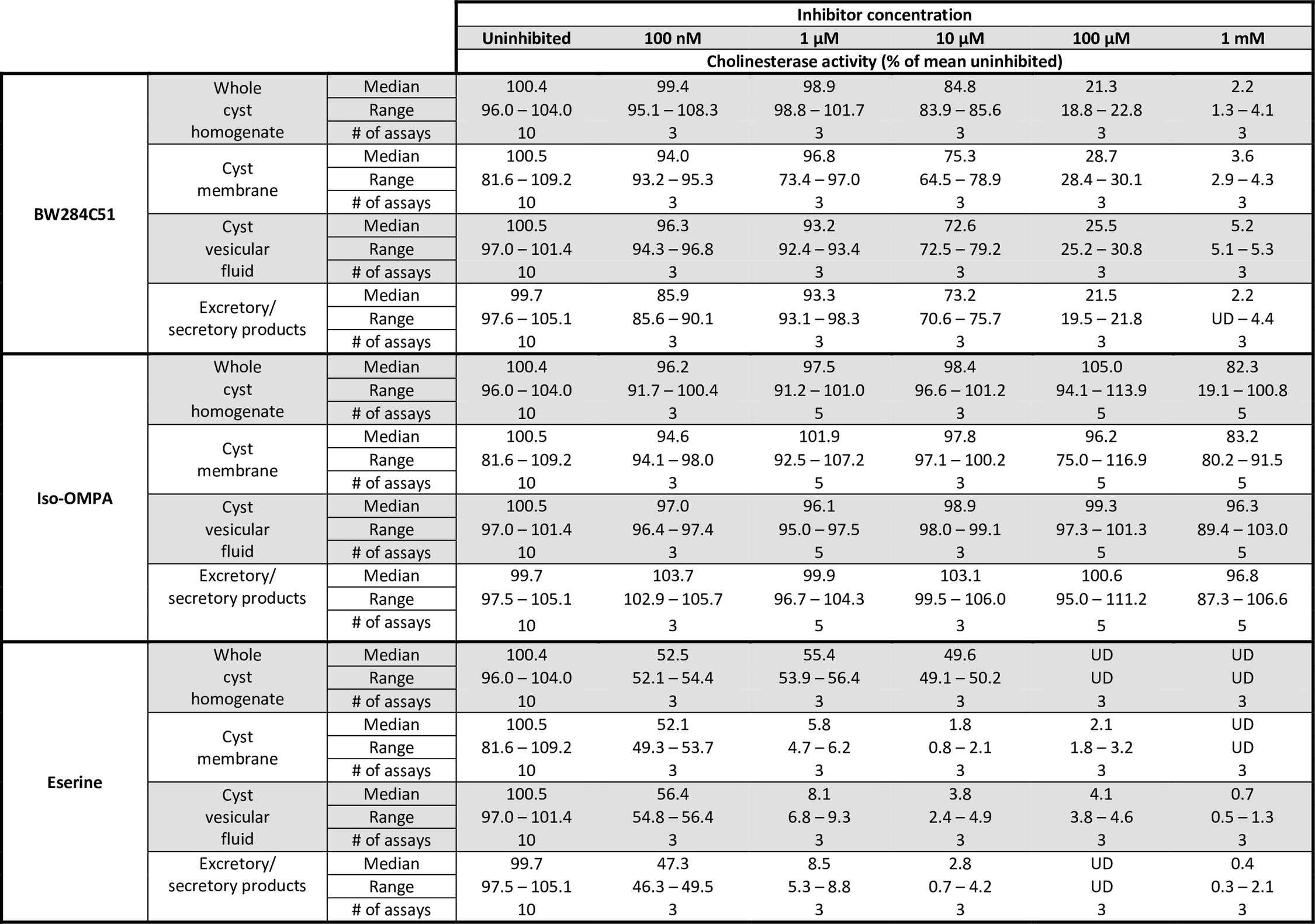

Supplement: S1 Table — Legend: UD = undetectable, where activity was so low as to be undetectable. (TIF) [file pntd.0008966.s001.tif]

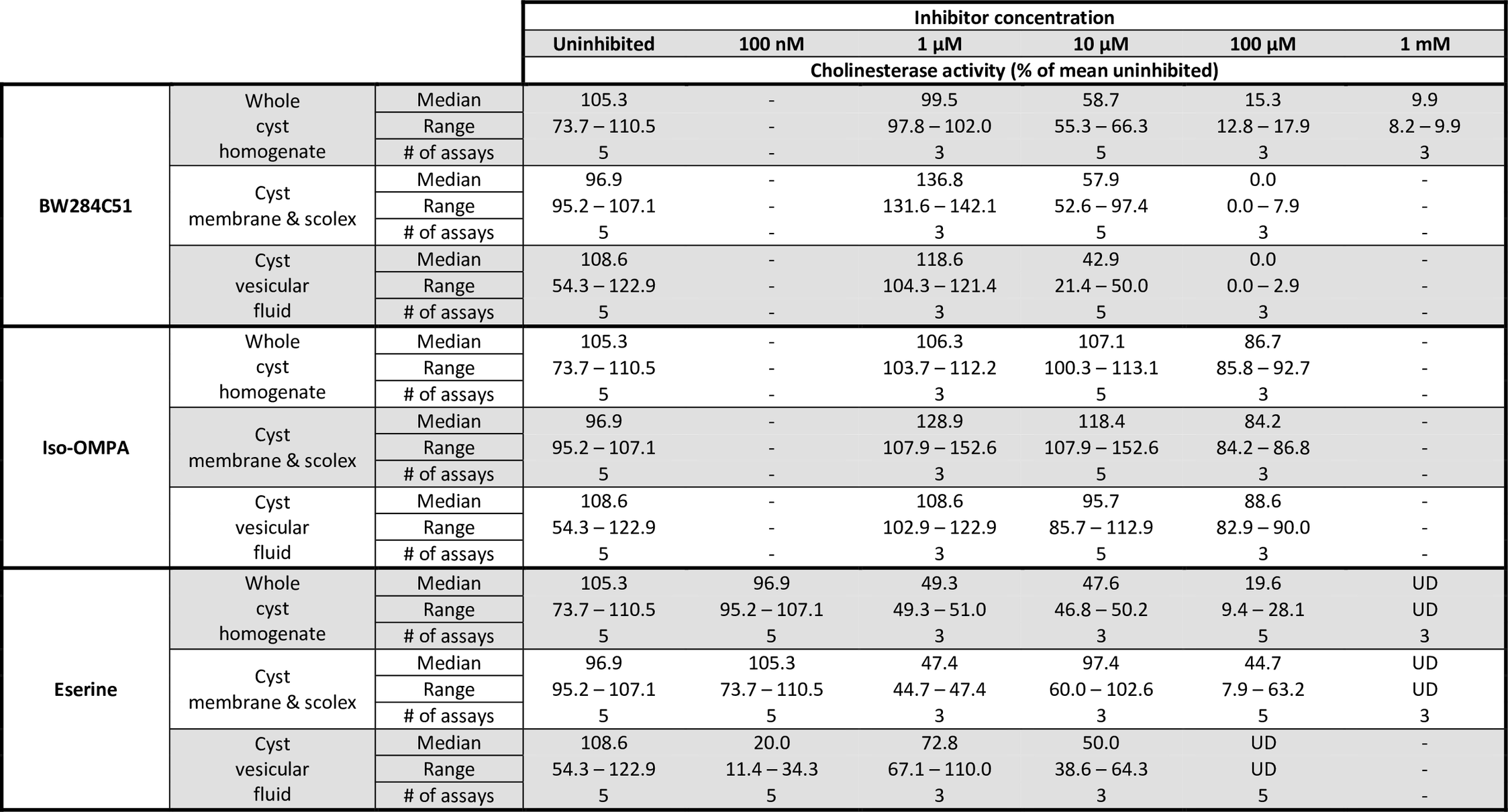

Supplement: S2 Table — Legend: UD = undetectable, where activity was so low as to be undetectable. (TIF) [file pntd.0008966.s002.tif]
